# CD8^**+**^ T-Cells Count in Acute Myocardial Infarction in HIV Disease in a Predominantly Male Cohort

**DOI:** 10.1155/2015/246870

**Published:** 2015-01-19

**Authors:** Oluwatosin A. Badejo, Chung-Chou Chang, Kaku A. So-Armah, Russell P. Tracy, Jason V. Baker, David Rimland, Adeel A. Butt, Adam J. Gordon, Charles R. Rinaldo, Kevin Kraemer, Jeffrey H. Samet, Hilary A. Tindle, Matthew B. Goetz, Maria C. Rodriguez-Barradas, Roger Bedimo, Cynthia L. Gibert, David A. Leaf, Lewis H. Kuller, Steven G. Deeks, Amy C. Justice, Matthew S. Freiberg

**Affiliations:** ^1^Division of General Internal Medicine, University of Pittsburgh, UPMC Montefiore Hospital, Pittsburgh, PA 15213, USA; ^2^Department of Biostatistics, University of Pittsburgh Graduate School of Public Health, Pittsburgh, PA 15261, USA; ^3^Yale School of Medicine, Yale University, New Haven, CT 06510, USA; ^4^University of Vermont, Burlington, VT 05446, USA; ^5^Department of Medicine, Hennepin County Medical Center, University of Minnesota, Minneapolis, MN 55415, USA; ^6^VA Medical Center, Emory University School of Medicine, Atlanta, GA 30033, USA; ^7^Division of Infectious Diseases, University of Pittsburgh School of Medicine, Pittsburgh, PA 15213, USA; ^8^VA Pittsburgh Healthcare System, Pittsburgh, PA 15240, USA; ^9^Hamad Medical Corporation, P.O. Box 3050, Doha, Qatar; ^10^Department of Pathology, University of Pittsburgh School of Medicine, Pittsburgh, PA 15213, USA; ^11^Department of Infectious Diseases and Microbiology, University of Pittsburgh Graduate School of Public Health, Pittsburgh, PA 15261, USA; ^12^Clinical Addiction Research and Education Unit, Boston University School of Medicine, Boston, MA 02118, USA; ^13^Boston University School of Public Health, Boston, MA 02118, USA; ^14^Department of General Internal Medicine and Public Health, Vanderbilt University Medical Center, Nashville, TN 37203, USA; ^15^Department of Medicine, VA Greater Los Angeles Healthcare System, Los Angeles, CA 90073, USA; ^16^Department of Medicine, David Geffen School of Medicine, Los Angeles, CA 90095, USA; ^17^Michael E. DeBakey VA Medical Center, Infectious Diseases Section, Baylor College of Medicine, Houston, TX 77030, USA; ^18^VA North Texas Healthcare System, Dallas, TX 75216, USA; ^19^Division of Infectious Diseases, University of Texas Southwestern Medical Center, Dallas, TX 75390, USA; ^20^Washington DC VA Medical Center, Washington, DC 20422, USA; ^21^George Washington University Medical Center, Washington, DC 20037, USA; ^22^Department of Epidemiology, University of Pittsburgh Graduate School of Public Health, Pittsburgh, PA 15261, USA; ^23^Department of Medicine, University of California, San Francisco, CA 94143, USA; ^24^Veteran Affairs Connecticut Health Care System, West Haven Veterans Administration Medical Center, Yale School of Medicine, Yale University, New Haven, CT 06516, USA; ^25^Cardiovascular Medicine Division, Vanderbilt University Medical Center, 2525 West End, Suite 300-A, Nashville, TN 37203, USA

## Abstract

Human Immunodeficiency Virus- (HIV-) infected persons have a higher risk for acute myocardial infarction (AMI) than HIV-uninfected persons. Earlier studies suggest that HIV viral load, CD4^+^ T-cell count, and antiretroviral therapy are associated with cardiovascular disease (CVD) risk. Whether CD8^+^ T-cell count is associated with CVD risk is not clear. We investigated the association between CD8^+^ T-cell count and incident AMI in a cohort of 73,398 people (of which 97.3% were men) enrolled in the U.S. Veterans Aging Cohort Study-Virtual Cohort (VACS-VC). Compared to uninfected people, HIV-infected people with high baseline CD8^+^ T-cell counts (>1065 cells/mm^3^) had increased AMI risk (adjusted HR = 1.82, *P* < 0.001, 95% CI: 1.46 to 2.28). There was evidence that the effect of CD8^+^ T-cell tertiles on AMI risk differed by CD4^+^ T-cell level: compared to uninfected people, HIV-infected people with CD4^+^ T-cell counts ≥200 cells/mm^3^ had increased AMI risk with high CD8^+^ T-cell count, while those with CD4^+^ T-cell counts <200 cells/mm^3^ had increased AMI risk with low CD8^+^ T-cell count. CD8^+^ T-cell counts may add additional AMI risk stratification information beyond that provided by CD4^+^ T-cell counts alone.

## 1. Introduction

Human Immunodeficiency Virus- (HIV-) infected persons have a higher risk for acute myocardial infarction (AMI) than HIV-uninfected persons [1, 2]. This excess risk is predicted in part by immune status in those with HIV infection [1, 3, 4]. During the course of untreated HIV infection, CD4^+^ T-cell counts decline. Among untreated and treated HIV-infected adults, lower CD4^+^ T-cell counts are associated with greater risk of comorbid disease [5] including AMI risk or subclinical coronary atherosclerosis [1, 3, 4, 6, 7]. Traditional cardiovascular disease (CVD) risk assessment tools like the Framingham Risk scores do not account for immune status and therefore may inaccurately estimate CVD risk in the setting of HIV [8]. Identifying additional prognostic biomarkers of CVD risk may be useful for CVD risk prediction in the setting of HIV.

The association between CD8^+^ T-cell counts and incident AMI risk remains largely unexplored [4]. In a nested case-control study of the French Hospital Database on HIV [4], Lang and colleagues found that a high current CD8^+^ T-cell count is associated with increased AMI risk, independent of cardiovascular risk factors and antiretroviral therapy. This study did not have an HIV-uninfected control group and did not consider additional potential confounders, such as hemoglobin concentration, renal function, and hepatitis C viral infection.

Total CD8^+^ T-cell counts are often obtained during routine care of HIV-infected persons and are used in the calculation of a CD4^+^/CD8^+^ T-cell ratio, which provides information on immune status beyond CD4^+^ counts alone. We assessed the association between routinely available total CD8^+^ T-cell count and the risk of AMI in a large cohort of HIV-infected and HIV-uninfected persons, adjusting for common traditional cardiovascular risk factors as well as HIV-specific parameters.

## 2. Materials and Methods

We examined the association between CD8^+^ T-cells and AMI risk among 73,398 persons enrolled in the U.S. Veterans Aging Cohort Study-Virtual Cohort (VACS-VC) [9] who were free of cardiovascular disease at baseline date (April 2003). Participants were followed through December 2009 for a mean follow-up period of 4.98 years. Details regarding this cohort have been published previously [1]. Among HIV-infected participants, 18,289 had available baseline CD8^+^ data at the time of enrollment of which 16,599 had both CD4^+^ and CD8^+^ data. There were 55,109 HIV-uninfected participants. The mean (±SD) age was approximately 48 (±9) years (HIV-infected) and 49 (±9) years (HIV-uninfected). Over 97 percent were men and 48 percent were African American. The outcome of interest was all incident AMI cases (nonfatal and fatal) in the VACS-VC that were completely managed in either VA or non-VA hospitals as previously described [1]. Briefly, incident AMI was defined using enzyme data, EKG charts, clinical data, 410 in-patient ICD-9 codes (Medicare), and/or death certificates.

The main independent variable of interest was the baseline total CD8^+^ T-cell count, which was analyzed separately as a continuous variable and categorically. CD8^+^ T-cell counts were available only for HIV-seropositive Veterans as they were obtained as part of routine clinical care. For the assessment as a continuous variable, the CD8^+^ T-cell count analysis was restricted to HIV-infected participants. As a categorical variable, study participants were classified as being either HIV-uninfected (the referent group) or HIV-infected with low, moderate, or high total CD8^+^ T-cells (based on tertiles). Using the same referent group, we then stratified these categories among HIV-infected people by baseline CD4^+^ T-cell count (≥500, 200–499, and <200 cells/mm^3^). The covariates included in the multivariable models were age, gender, race, high blood pressure (controlled/uncontrolled), diabetes, triglyceride levels, high density lipoprotein levels, low density lipoprotein levels, body mass index, smoking history, hepatitis C virus infection, estimated glomerular filtration rate, statin use, hemoglobin concentration, cocaine and alcohol abuse, and/or dependence as previously described [1]. We included missing covariate data in our analyses using multiple imputation techniques that generated five data sets with complete covariate values to increase the efficiency and robustness of the estimated hazard ratios. Stata version 12 was used for all statistical analyses and a *P* value of <0.05 was considered to indicate statistical significance.

## 3. Results and Discussion

**Figure 1:**
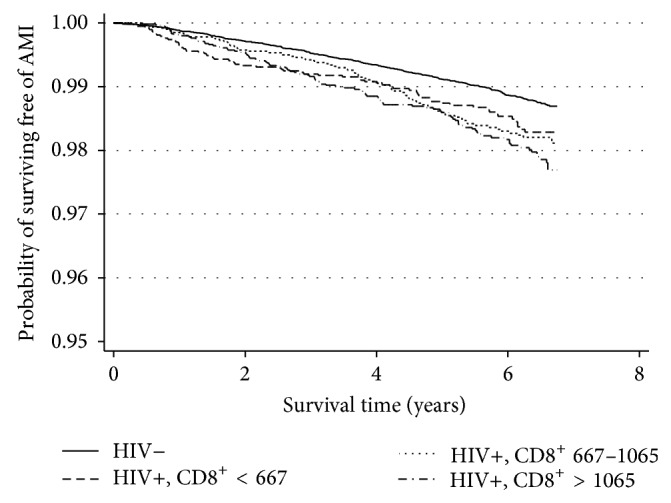
Graph of Kaplan-Meier survival estimates of AMI development for different HIV/CD8^+^ T-cell strata. Abbreviations used are HIV−, HIV-uninfected, HIV+, HIV-infected. All T-cell counts in cells/mm^3^. Difference in survival function equality based on Tarone-Ware and Peto-Peto-Prentice tests had a *P* value <0.001, *df* = 3.

Survival free from AMI was different among HIV-uninfected and the three tertiles of HIV-infected people (*P* value <0.001, Figure 1). The poorest survival free of AMI was observed among those in the highest CD8^+^ T-cell tertile (>1065 cells/mm^3^). Increasing CD8^+^ T-cell counts were associated with a modest increase in AMI risk among HIV-infected people (HR per 100 CD8^+^ T-cells (95% CI): 1.03 (1.01–1.05); Table 1). Compared to uninfected participants, HIV-infected participants in all tertiles of CD8^+^ T-cell count had a significantly increased risk of AMI. There was a stepwise increase in AMI risk with increasing CD8^+^ T-cell count with the highest risk in the highest CD8^+^ T-cell tertile (>1065 cells/mm^3^; HR = 1.82, 95% CI: 1.46–2.28; Table 1). We found evidence that the effect of CD8^+^ T-cell tertiles on AMI risk differed by CD4^+^ T-cell level. Compared to uninfected people, HIV-infection with a high CD8^+^ T-cell count was associated with AMI among those with CD4^+^ T-cell counts ≥200 cells/mm^3^ while a low CD8^+^ T-cell count was associated with a higher risk of AMI among those with CD4^+^ T-cell counts <200 cells/mm^3^ (Table 1). The stepwise increase in AMI risk with increasing CD8^+^ T-cell count was most extreme among HIV-infected people with CD4^+^ T-cell counts between 200 and 499 cells/mm^3^ (Table 1).

In summary, while increasing CD8^+^ T-cell count was associated with increasing risk of AMI, stratification by CD4^+^ T-cell count unmasked potentially important associations: higher CD8^+^ T-cell counts are associated with AMI risk in those with CD4^+^ T-cell counts ≥200 cells/mm^3^ while lower CD8^+^ T-cell counts are associated with AMI risk in those with low CD4^+^ T-cell counts. Second, the impact of a high versus low CD8^+^ T-cell count was most evident in those individuals with CD4^+^ T-cell counts of 200–499 cells/mm^3^. Importantly, all-cause mortality rates were similar across all three CD8^+^ T-cell tertiles among those with CD4^+^ T-cell counts ≥200 cells/mm^3^.

Among participants for whom high CD8^+^ T-cell counts predicted AMI (i.e., CD4^+^ T-cell count ≥200 cells/mm^3^), it is possible that activation of the large numbers of CD8^+^ T-cells may contribute to vascular damage [10]. In contrast, our finding that HIV-infected Veterans with a low CD4^+^ T-cell level appeared to be at greatest AMI risk when their CD8^+^ T-cell count was also low is consistent with earlier work suggesting that AMI risk is linked to immunodeficiency [1, 3]. Decline in both CD4^+^ and CD8^+^ T-cell counts is a manifestation of very advanced disease and likely reflects loss of regenerative potential (e.g., loss of hematopoietic stem cells) [11]. Moreover, decline of the adaptive immune system may increase innate immune activity, which could also increase AMI risk [12].

The study had limitations. First, we did not explore changes in HIV-specific CD8^+^ T-cell counts nor did we examine markers for CD8^+^ T-cell immune activation, such as CD38^+^. Time-updated covariates for CD8^+^ (and CD4^+^) T-cells were not used to determine AMI risk. Third, over 90% of the participants were men (~89% were men in the study by Lang et al. [4]), so the study results may not be generalizable to women. All-cause mortality rates were highest among those with CD4^+^ T-cell counts <200 cells/mm^3^; therefore AMI risk may be underestimated due to competing risk of death. Finally, our clinical data does not allow further delineation of T-cell subsets that have been associated with atherosclerotic (e.g., T helper 1 (T_H_1) cells) and antiatherosclerotic processes (e.g., regulatory T helper 2 (T_H_2) cells) [12].

## 4. Conclusions

In conclusion, high CD8^+^ T-cell count among HIV-infected people was associated with increased acute myocardial infarction risk compared to uninfected people. The association between CD8^+^ T-cell count and AMI appears to differ by CD4^+^ T-cell count. CD8^+^ T-cell count may add additional AMI risk stratification information beyond that provided by CD4^+^ T-cell counts particularly among those with CD4^+^ T-cell counts between 200 and 499 cells/mm^3^. These findings should be confirmed in future studies with data on CD8^+^ T-cell counts among uninfected people and HIV-specific CD8^+^ T-cell counts.

## Figures and Tables

**Table 1:** Acute myocardial infarction rates and risk and all-cause mortality rates by HIV status, CD8^+^ T-cell count, and CD4^+^ T-cell strata.

Regression model^a^	Independent variable^b^	*N* (% of HIV+)^c^	AMI rate (95% CI)^d^	HR (95% CI)	*P *value	Mortality rates (95% CI)^q^
I	Per 100 CD8^+^cells (HIV+ only)	18,289		1.03 (1.01–1.05)	0.006	

II	HIV-uninfected	55,109	18.49 (16.95–20.17)	1.00	Ref	18.63 (18.10–19.17)
	HIV+ All CD4^+^ strata	**18,289**				
	CD8^+^ < 667	5,987 (32.74)	26.08 (20.70–32.86)	1.45 (1.12–1.88)^e^	0.005	63.17 (60.00–66.51)
	CD8^+^ 667–1065	6,185 (33.82)	26.98 (21.81–33.37)	1.54 (1.21–1.96)^f^	<0.001	38.54 (36.23–41.00)
	CD8^+^ > 1065	6,117 (33.45)	32.20 (26.50–39.14)	1.82 (1.46–2.28)^g^	<0.001	40.89 (38.49–43.45)

III	HIV-uninfected	55,109	18.49 (16.95–20.17)	1.00	Ref	18.63 (18.10–19.17)
	HIV+ CD4^+^ ≥ 500	**5,422**				
	CD8^+^ < 667	1,097 (20.23)	24.00 (14.70–39.18)	1.30 (0.76–2.20)^h^	0.339	28.08 (24.05–32.78)
	CD8^+^ 667–1065	1,971 (36.35)	26.68 (18.76–37.93)	1.51 (1.03–2.21)^i^	0.037	24.83 (21.89–28.17)
	CD8^+^ > 1065	2,354 (43.42)	28.68 (20.96–39.26)	1.69 (1.21–2.36)^j^	0.002	30.66 (27.57–34.10)
	HIV+ CD4^+^ 200–499	**6,730**				
	CD8^+^ < 667	1,901 (28.25)	21.54 (14.32–32.42)	1.22 (0.80–1.87)^k^	0.360	43.88 (39.72–48.48)
	CD8^+^ 667–1065	2,447 (36.36)	26.08 (18.81–36.16)	1.47 (1.03–2.09)^l^	0.034	37.68 (34.27–41.42)
	CD8^+^ > 1065	2,382 (35.40)	37.38 (28.25–49.46)	2.08 (1.53–2.82)^m^	<0.001	43.52 (39.74–47.67)
	HIV+ CD4^+^ < 200	**4,447**				
	CD8^+^ < 667	2,389 (53.72)	32.16 (22.86–45.23)	1.82 (1.26–2.64)^n^	0.001	107.13 (100.36–114.35)
	CD8^+^ 667–1065	1,171 (26.33)	29.60 (18.65–46.97)	1.80 (1.10–2.94)^o^	0.019	67.40 (60.56–75.02)
	CD8^+^ > 1065	887 (19.94)	27.89 (16.19–48.02)	1.51 (0.85–2.67)^p^	0.158	63.32 (55.81–71.85)


^a^The covariates included in the multivariable models (hazard ratios not shown) were age, gender, race, high blood pressure (controlled/uncontrolled), diabetes, triglyceride levels, high density lipoprotein levels, low density lipoprotein levels, body mass index, smoking history, hepatitis C virus infection, estimated glomerular filtration rate, statin use, hemoglobin concentration, cocaine and alcohol abuse, and/or dependence.

^b^CD8^+^ and CD4^+^ T-cell counts were measured in cells/mm^3^.

^c^While all 18,289 HIV-infected participants had baseline CD8^+^ T-cell count measurements, 1,690 of them lacked baseline CD4^+^ counts. Thus, these persons were excluded from analyses involving both CD4^+^ and CD8^+^ T-cell counts.

^d^AMI rates were measured per 10,000 person years.

^e versus f^ *P* value comparing these hazard ratios was <0.001.

^e versus g^ *P* value comparing these hazard ratios was <0.001.

^f versus g^ *P* value comparing these hazard ratios was <0.01.

^h versus i^ *P* value comparing these hazard ratios was 0.026.

^h versus j^ *P* value comparing these hazard ratios was <0.001.

^i versus j^ *P* value comparing these hazard ratios was <0.001.

^k versus l^ *P* value comparing these hazard ratios was 0.092.

^k versus m^ *P* value comparing these hazard ratios was <0.001.

^l versus m^ *P* value comparing these hazard ratios was <0.001.

^n versus o^ *P* value comparing these hazard ratios was 0.002.

^n versus p^ *P* value comparing these hazard ratios was <0.004.

^o versus p^ *P* value comparing these hazard ratios was <0.066.

^q^All-cause mortality rates were measured per 10,000 person years.
